# Kidney bean detection in complex agricultural scenarios based on the kidneyB-YOLO model

**DOI:** 10.3389/fpls.2025.1672179

**Published:** 2025-12-12

**Authors:** Jianjia Qi, Chunxiang Liu, Hongxia Chu

**Affiliations:** College of Mechanical and Electrical Engineering, Heilongjiang Institute of Technology, Harbin, China

**Keywords:** precision agriculture, object detection, model comparison, agricultural scenarios, deep learning

## Abstract

**Introduction:**

This study addresses the problem of detecting multiple targets and occluded kidney beans in the complex agricultural scenarios under the open-field trellis cultivation.

**Methods:**

A target detection model based on kidneyB-YOLO is proposed. The model integrated the dynamic convolution module, the Deformable Attention Transformer (DAT) attention mechanism, the feature fusion detection head improved Adaptively Spatial Feature Fusion (ASFF) detection method, and the Focaler-SIoU loss function. The dynamic convolution module adaptively adjusted parameters based on the input. The DAT attention mechanism introduced a deformable attention mechanism, focusing only on a small key region of the image. The feature fusion detection head ASFF, an adaptive spatial feature fusion method, filtered out conflicting target information. The new loss function module, Focaler-SIoU, was formed by combining the SIoU loss function with the Focaler-IoU algorithm. An open-canopy environment bean pod dataset was constructed for model training and validation.

**Results:**

The results showed that the KidneyB-YOLO model exhibited significant improvements compared to the original YOLOv8n, especially in occluded scenarios. The model achieved a detection performance of 85.90% mAP with a computational complexity of 12.4 GFLOPs, a model size of 12 MB, and operated at 32.5 FPS.

**Discussion:**

The enhancements improved the accuracy and robustness of bean pod detection. The model demonstrated strong robustness and generalization capability in the fruit detection task for kidney bean harvesting under open-air trellises.

## 1 Introduction

Kidney bean is an annual herbaceous plant in the legume family. It is relatively sensitive to light and grows best in deep, well-drained loamy soils. As a tender, flavorful, and nutritious vegetable, kidney beans are favored by a wide range of consumers. With the growing demand for higher vegetable yield and quality, the current kidney bean cultivation models struggle to produce high-quality products. The traditional open-field cultivation model faces challenges in achieving fine field management, yield monitoring, quality control, and automated harvesting, which makes the implementation of intelligent management difficult (Ilić and Fallik, 2017; Chen et al., 2024). As a climbing crop, kidney beans require support structures during their growing period. In modern agricultural production, the detection and segmentation of kidney bean fruits are essential for the intelligent management of their growth. Developing accurate and reliable detection models based on open-field trellis cultivation holds significant research value and practical application. In an open-field trellis environment, the fruits and stems of the kidney bean plant are often heavily obstructed; moreover, the fruits, stems, and leaves have similar coloration, making them difficult to distinguish. The complex background of open-field environments further complicates image analysis (Xiao et al., 2023).

Early methods of fruit identification relied upon traditional image processing techniques and machine learning algorithms (Weng et al., 2025; Xie et al., 2025); they achieved relatively good results under simple conditions, but they failed to effectively identify fruits. With the development of computer vision technology, deep learning models have achieved significant advancements in image segmentation (Gao et al., 2025; Tariq et al., 2025). State-of-the-art YOLO series models have become the most widely applied models in the field of object detection. As a single-stage detection method, YOLO directly realizes object classification and boundary box localization, making it particularly suitable for real-time applications due to its speed and efficiency (Li et al., 2025; Lv et al., 2025). YOLOv8, as a single-stage object detection algorithm in the YOLO series, converts object detection into a regression problem, thereby directly identifying the category and position information of radishes and realizing precise object detection (Yu et al., 2025).

The detection of occluded and small objects represented one of the most challenging frontier issues in the field of computer vision and object detection. When “small objects” and “severe occlusion” occurred simultaneously, the complexity of the problem increased exponentially, posing an extreme challenge to existing detection models. In recent years, certain achievements have been made through improvements and optimizations based on YOLO for fruit detection in complex environments. Numerous researchers (Chen et al., 2023b; Zhu et al., 2024; Jin et al., 2025) constructed various camellia oleifera fruit datasets and enhanced YOLO models from different perspectives to achieve accurate identification of camellia fruits. Additionally, researchers proposed solutions for the recognition of tomatoes (Li et al., 2022; Liang et al., 2025), apples (Jiuxin et al., 2023; Kaukab et al., 2024), lychees (Li et al., 2024a), goji berries (Wang et al., 2024), and other fruits characterized by small sizes and frequent occlusion. Dong et al. (2024) addressed the challenge of accurately detecting cherries entwined with foliage occlusions and fruit overlaps by developing the SFF-YOLOv5s model, which was based on the YOLOv5s framework. By incorporating the Coordinate Attention (CA) mechanism into the trunk network, they enhanced the model’s ability to extract critical fruit information. This study substituted the Similarity IoU (SIoU) loss function for the Close Image IoU (CIoU) loss function, thereby improving the detection accuracy of the model. Ge et al. (2025) improved the YOLOv8n model to address the challenges of tomato object detection in complex agricultural environments. They utilized a spatial pyramid pooling enhanced with an ELAN (SPPELAN) structure to replace the original spatial pyramid pooling fast (SPPF) structure, minimizing information loss during the convolution processes. Additionally, they introduced the partial self-attention (PSA) attention mechanism into the feature extraction stage without adding any computational burden. These modifications improved the model’s robustness and generalization capabilities. Ren et al. (2025) implemented a lightweight detection approach for apple roses under different cultivation patterns by improving the YOLOv8n model. To address the issues of poor accuracy, missed detections, and false positives in fruit detection and localization, they proposed a lightweight detection method, denoted as YOLO-iBPD. This method re-placed the main trunk module, C2f, in the YOLOv8n framework to enhance the model’s feature extraction and expression capabilities. Additionally, they modified the boundary box loss function to PIoUv2 to better focus on extracting fruit-related information. The model demonstrated improved stability and robustness under the constraint of light-weight design. Wang et al. (2025a) improved the YOLOv8n model for quality detection of apple surface appearance under natural scenes. To enhance the model’s ability to handle complex environments with multi-occlusion problems, they incorporated a combination of partial convolution (PConv) and replaced the main trunk module, C2f, in the YOLOv8n framework, while adding an efficient multiscale attention (EMA) mechanism to improve the model’s attention capabilities at various scales. However, the improvements in terms of accuracy, recall, and average precision compared to YOLOv8n were limited to 3.4%, 1.1%, and 1.3%, respectively, indicating room for further enhancement. Additionally, despite extensive research on fruit target detection techniques, most studies did not consider the complex open-air-covered scenarios, where the fruits and stems can be occluded by each other and it challenging to accurately identify the fruit boundaries due to their simi-lar background colors (Tang et al., 2023).

It is noteworthy that the development of lightweight models balancing accuracy and efficiency has become a significant trend in the field of agricultural object detection. In recent years, researchers have proposed various efficient architectural designs to adapt to resource-constrained deployment environments. For instance, Sun (2024)) proposed the S-YOLO model for greenhouse tomato detection, which achieved a balance between high accuracy and fast inference through lightweight designs such as depthwise separable convolutions. Wang et al. (2025b) introduced LSOD-YOLO specifically for lightweight small object detection, optimizing the feature fusion pathway to enhance sensitivity to small targets. For walnut blockage detection, Wang et al. (2025c) developed Ow-YOLO by pruning and restructuring modules of YOLOv8s, significantly reducing model size and computational cost while maintaining accuracy. Zhang and Jiang (2025) designed AHN-YOLO to address the challenge of detecting dense, small-sized tomatoes, improving detection performance for tiny fruits by incorporating an efficient attention mechanism and a lightweight neck network. Zhong et al. (2024) proposed the Light YOLO model specifically for mango detection, which employed a lightweight backbone and a simplified feature pyramid structure to greatly increase detection speed while maintaining high accuracy. Collectively, these works have advanced the application depth of lightweight models in agricultural vision tasks.

However, although previous studies conducted extensive research on fruit detection technology, most experiments did not involve complex scenarios such as open-air trellis environments. In such settings, the severe mutual occlusion between fruits and stems/leaves, coupled with the high color resemblance between fruits and the background, posed significant challenges for accurate fruit boundary identification. Existing general models often misinterpreted a single fruit as multiple fragments due to occlusion during detection. For elongated fruit like kidney beans, partial occlusion—while not affecting human perception of their continuous position and appearance—seriously impaired visual models’ ability to uniformly recognize and precisely localize the complete target.

Therefore, we propose a method capable of adapting to varying degrees of occlusion and complex backgrounds in open-field trellis cultivation scenarios for kidney bean target recognition. The method integrates a dynamic convolution (DynamicConv) mechanism combined with the original C2f module to form a dynamic convolution module, which incorporates an attention mechanism Deformable Attention Transformer and adds an improved adaptive spatial feature fusion (ASFF) detection head. Additionally, we design the Focaler-SIoU loss function to enhance computational efficiency, thereby refining feature extraction. This study provided a technical approach for target detection of fresh-vegetable fruits such as kidney beans that require open-air trellis cultivation, demonstrating excellent robustness and generalization capability in the fruit detection task for kidney bean harvesting under open-air trellises.

## 2 Materials and methods

### 2.1 Construction of the dataset

#### 2.1.1 Data collection

Mature kidney bean fruits cluster densely on a single vine, making the construction of diverse sample datasets under various conditions critical for improving a model’s robustness and generalization capabilities. To ensure the model achieves good generalization ability and robustness, the dataset must include samples with varying sizes, resolutions, overlapping fruits, complex backgrounds, illumination variations, and distance disturbances, all of which aim to enhance the model’s generalization ability in real-world agricultural scenarios and improve accuracy and stability in practical applications (Chen et al., 2023a; Jia et al., 2025). Based on the aforementioned requirements and the data collection approach of subsequent automated harvesting robots, this study constructed a sample dataset using screenshots from videos.

With a handheld camera, we captured three vertical heights and three horizontal distances, recording four segments of video footage at a rate of one image per second. The dataset consists of 836 original kidney bean images in JPG format, featuring varying resolutions and aspect ratios to meet the non-standard task requirements of target recognition in real-world agricultural scenarios. The dataset encompassed various lighting conditions, including morning, noon, evening, and cloudy weather, as well as multiple occlusion scenarios such as no occlusion, partial occlusion, and heavy occlusion. It also incorporated complex backgrounds featuring open-air metal trellises and included images with varying degrees of fruit overlap. Although the significant variation in image resolutions increased the difficulty of model training, it contributed to enhancing the model’s adaptability and robustness in diverse real-world scenarios. During the annotation process, only fully visible kidney bean instances were labeled. Targets with occlusion exceeding approximately 50% of the fruit body were excluded from annotation. Bounding boxes were strictly drawn to cover only the visible parts, without inferring occluded regions, following common annotation standards for handling occlusion (Mavridou et al., 2019). The construction of this dataset took into account key factors affecting kidney bean detection in real agricultural environments, ensuring its close alignment with practical scenarios. The dataset was constructed with a comprehensive consideration of factors critical to real-world agricultural scenarios, ensuring good alignment with real-world agricultural environments.

#### 2.1.2 Dataset expansion

**Figure 1:**
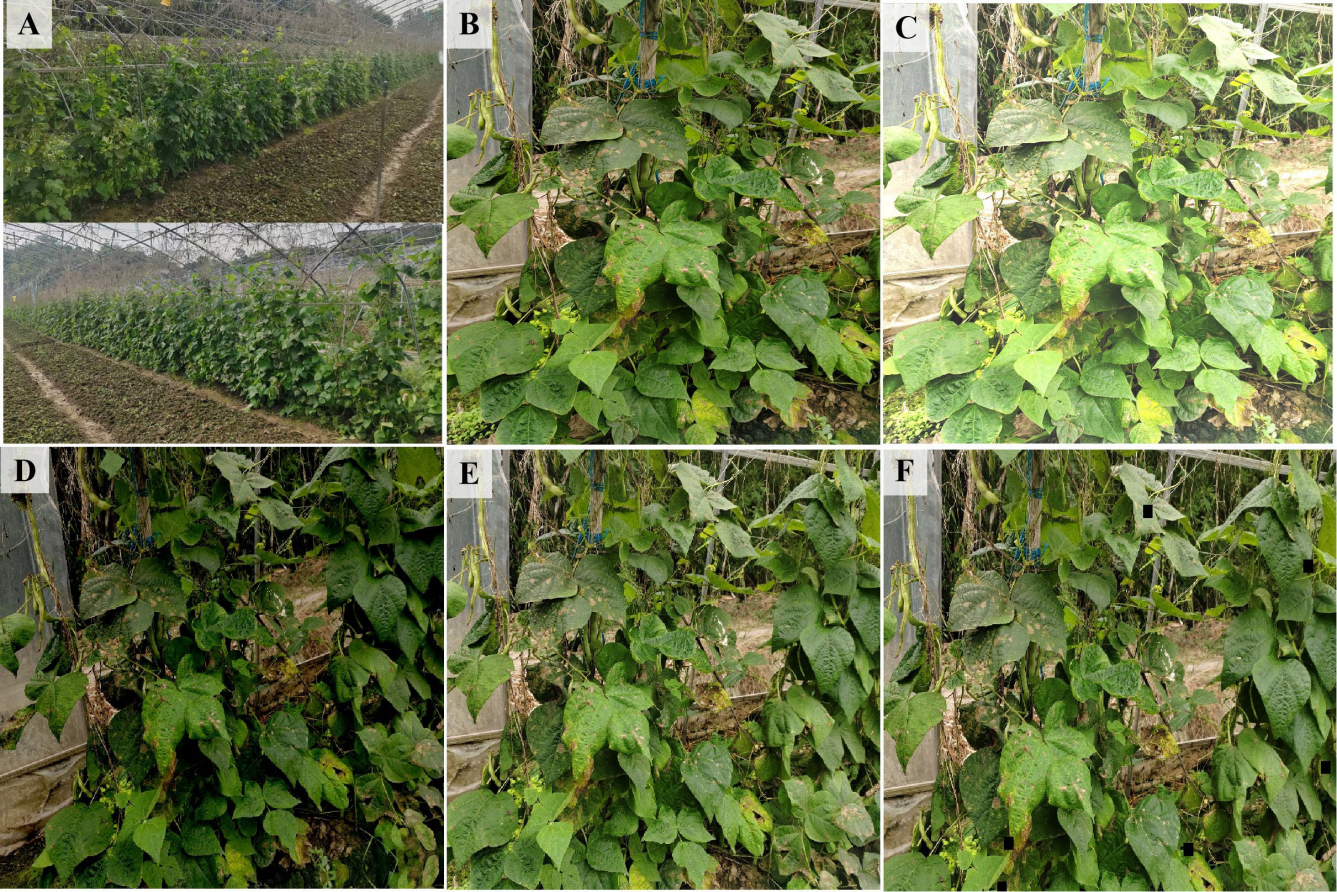
Open-field trellis cultivation environment and examples of the various approaches to dataset augmentation. **(A)** Open-field trellis cultivation environment, **(B)** Original image, **(C)** Brightness and contrast adjustment, **(D)** Gaussian blur and secure clipping, **(E)** Rotation and secure cutting, **(F)** Covering and secure cropping.

To further enrich the feature content of the self-built kidney bean dataset and expand its scale, data augmentation techniques were employed. After data augmentation, the dataset enabled the model to learn more effectively from complex natural environments, thereby enhancing its generalization capability in challenging background scenarios (Ma et al., 2024). The augmented dataset contains more than 3000 sample images. These images are randomly divided into training, validation, and test sets in a 7:2:1 ratio. **Figure 1** demonstrates that various augmentation techniques, including increasing or decreasing the brightness, applying Gaussian blur, performing safe cropping, and rotating the images, were applied to the training and validation sets. (B) Original image, (C) Brightness and contrast adjustment, (D) Gaussian blur and secure clipping, (E) Rotation and secure cutting, (F) Covering and secure cropping. And **Figure 1**. (A) depicts the real-world open-field trellis cultivation scenario from which we collected the kidney bean images.

### 2.2 Construction of the kidney bean detection model

To balance the performance of object detection and computational resource constraints, the YOLOv8 series includes n/s/m/l/x specifications (Varghese and Sambath, 2024). As the model complexity increases exponentially during the extension process, the computational complexity of the detection process also increases exponentially. This significantly improves the accuracy of object detection but leads to an increase in training time, reduced efficiency in storage, and a decline in detection speed. These factors make it difficult to meet the deployment requirements and the frame rate demands for real-time detection of kidney beans in agricultural settings. To ensure that the model parameters and dimensions re-main within deployment thresholds while maintaining detection requirements, a balance must be struck. YOLOv8n, being the smallest model in the series, offers extremely fast inference speeds and high detection accuracy, making it suitable as a foundational frame-work for real-time kidney bean detection. However, in complex agricultural environments, factors such as leaf occlusion, fruit overlaps, and complex backgrounds significantly degrade detection precision, resulting in high rates of false negatives and false positives. Consequently, YOLOv8n demonstrates limited robustness and detection accuracy in real world scenarios and fails to meet the demands of field operations. YOLOv8n-p2, which is specifically designed for detecting small targets with multiple occlusions, addresses some of these challenges (Jia et al., 2025). In order to improve the detection accuracy and meet the requirements for the real-time detection of kidney beans in field operations, we selected YOLOv8n-p2 as the foundation framework for optimizing the detection of fruit targets in the open-field trellis cultivation environment of kidney bean growing. Through gradually increasing the structural complexity while ensuring higher detection accuracy, we introduced dynamic convolution modules, attention mechanisms, and feature fusion strategies to improve the model’s recognition of interference targets. Additionally, we optimized the loss function to enhance the similarity between detection boxes and predicted boxes, thereby improving the model’s performance in complex scenarios. The improved YOLOv8n-p2 model is named KidneyB-YOLO, reflecting its tailored design for kidney bean fruit detection.

#### 2.2.1 Kidney bean detection model based on the improved YOLOv8n-p2

**Figure 2:**
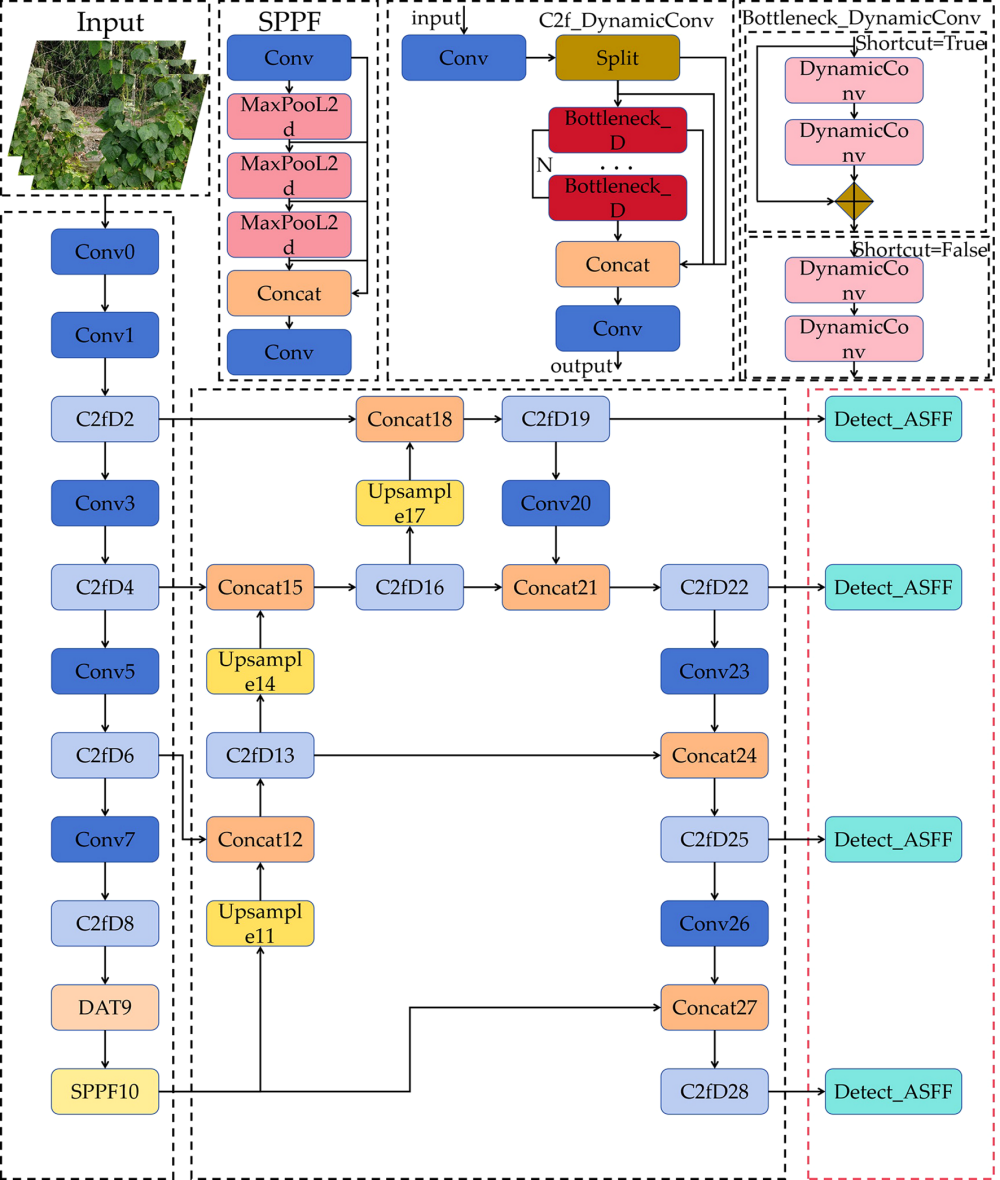
Architectural overview of the KidneyB-YOLO framework.

Based on YOLOv8n-p2, we combined the specific characteristics of kidney bean fruit detection in complex agricultural scenes to restructure the network architecture. Four key optimizations were implemented to enhance detection accuracy and performance: (1) DynamicConv was integrated by combining the original C2f module to form a dynamic convolution module, thereby minimizing information loss during convolution processes. (2) An attention mechanism, DAT, was added to increase the model’s focus on regions of interest while simultaneously maintaining performance without a significant increase in computational complexity. (3) An ASFF detection head was incorporated, along with the addition of a fourth detection head specifically designed for small target detection, to im-prove the precision of small target recognition. (4) A novel loss function, Focaler-SIoU, was developed to optimize the similarity between detection boxes and predicted boxes, thereby enhancing the model’s performance in complex scenarios. The overall framework of the improved model, KidneyB-YOLO, is illustrated in **Figure 2**.

#### 2.2.2 Construction of the dynamic convolution module

The C2f module exhibits issues such as computational redundancy and inadequate feature representation during feature extraction (Li et al., 2024b). To address these challenges and enhance the model’s ability to handle complex scenes and small targets, we improved the C2f module in the original YOLOv8n-p2 model by introducing DynamicConv. This mechanism was integrated into large-scale visual pretrained models to increase the number of parameters while aiming to minimize floating-point operations. As a result, the network maintains low floating-point operations but has increasing parameter quantities, thereby enabling the models to leverage features from the large-scale visual pre-trained models (Han et al., 2024).

DynamicConv operates by dynamically selecting or combining different convolution kernels (referred to as “experts”) for each input sample, allowing the network to adaptively adjust its parameters based on the input characteristics. This approach extends traditional convolution operations, enabling the network to self-adaptively adjust its architecture. In-stead of using fixed convolution kernels for all inputs, dynamic convolution employs multiple kernels (or parameter sets) that dynamically select the appropriate kernel based on the input’s properties. The selection process is governed by a learned function, such as a multi-layer perceptron (MLP) combined with a softmax function, which dynamically generates the weights controlling the contributions of each convolutional kernel. Given an input feature and a set of convolutional kernels, each kernel corresponds to an expert. The contribution of each expert is controlled by a dynamic coefficient, which is independently generated for each input sample. The output is the weighted sum of all dynamically selected convolutional operations. As shown in Equation 1 this dynamic approach allows the network to process inputs more efficiently while maintaining high accuracy, leveraging the flexibility of dynamic kernels to enhance feature extraction and model performance.

(1) $$\text{Y}=\sum_{i=1}^{\text{M}}{\text{α}}_{\text{i}}(\text{X}*{\text{W}}_{\text{i}})$$

where “ *“ denotes the convolution operation. ${\text{α}}_{\text{i}}$ is dynamically generated through a lightweight subnetwork whose input features are derived from global average pooling.

**Figure 3:**
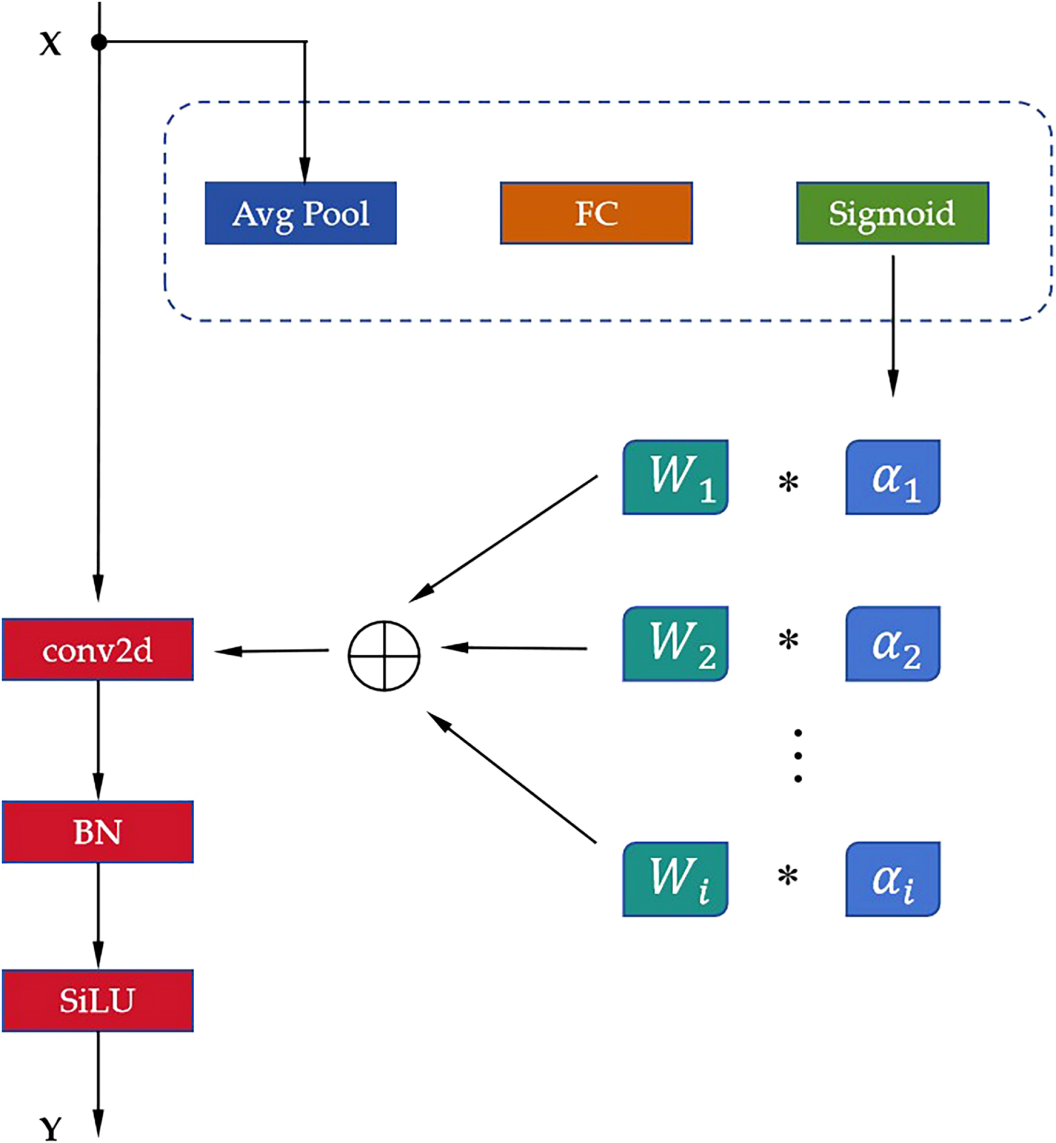
The structural principles of DynamicConv. "*" denotes the convolution operation.

DynamicConv offers several advantages. First, by employing shared and dynamically combined convolution kernels, this mechanism allows for a significant increase in model parameters while incurring minimal additional computational costs. Second, it enables the model to better adapt to diverse input features, thereby enhancing its generalization capability. Third, it permits the deployment of more complex network structures in resource-constrained environments without significantly increasing computational demands. The structural principle of DynamicConv is illustrated in **Figure 3**.

We integrated DynamicConv into the C2f module of the original YOLOv8n-p2 model. The C2f module is essentially a convolution block that extracts fundamental features from the input image through an initial convolution block and further refines and enhances these features using multiple Bottleneck blocks. These Bottleneck blocks are capable of capturing more complex patterns and details. The concatenated block is then used to fuse the directly transmitted feature maps and the processed feature maps, enabling the model to integrate multiscale and multilevel information comprehensively. Finally, the convolution block generates the ultimate feature map, providing rich feature representations for subsequent detection and classification tasks. However, the original C2f module, when used for kidney bean object detection in complex agricultural scenes, requires more floating-point operations. This leads to an increased computational burden without guaranteeing computational precision. To address this, we incorporated DynamicConv into the down-sampling module, integrating it with the C2f module to form the C2f_DynamicConv module. The principle of this new module enables the use of minimal computational resources to obtain more kidney bean spatial positions, sizes, and other detailed features. This enhances the model’s ability to perceive kidney bean characteristics and improves recognition accuracy.

#### 2.2.3 Self-attention mechanism

**Figure 4:**
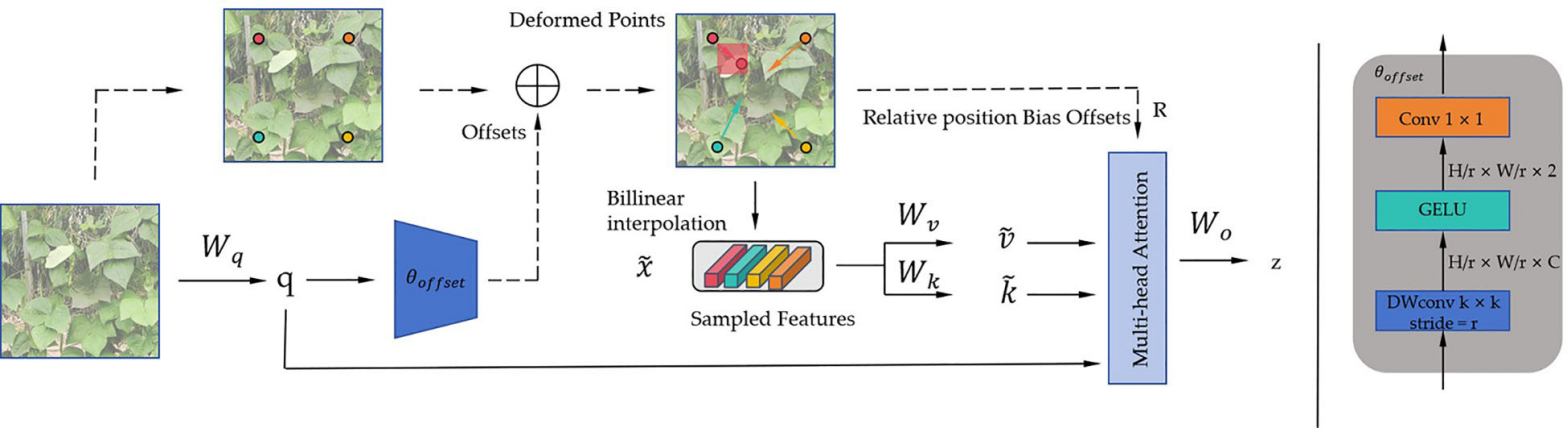
Architecture of the proposed DAT mechanism.

We added the DAT attention mechanism to the original model (Xia et al., 2022). DAT is a vision transformer that introduces a deformable attention mechanism. Traditional transformers employ standard self-attention mechanisms that process all pixels in an image, leading to high computational costs. DAT introduces the deformable attention mechanism, which focuses only on a small portion of key regions in the image. This approach significantly reduces computational costs while maintaining good performance. Within the DAT, sampling points are dynamically selected rather than the entire image being processed. This dynamic selection mechanism enables the model to concentrate on the regions that are most important for the current task. This can enhance global feature interactions, reduce information diffusion, and emphasize the representation of important information. The structure principle of the DAT mechanism is illustrated in **Figure 4**.

#### 2.2.4 Feature fusion detection head

**Figure 5:**
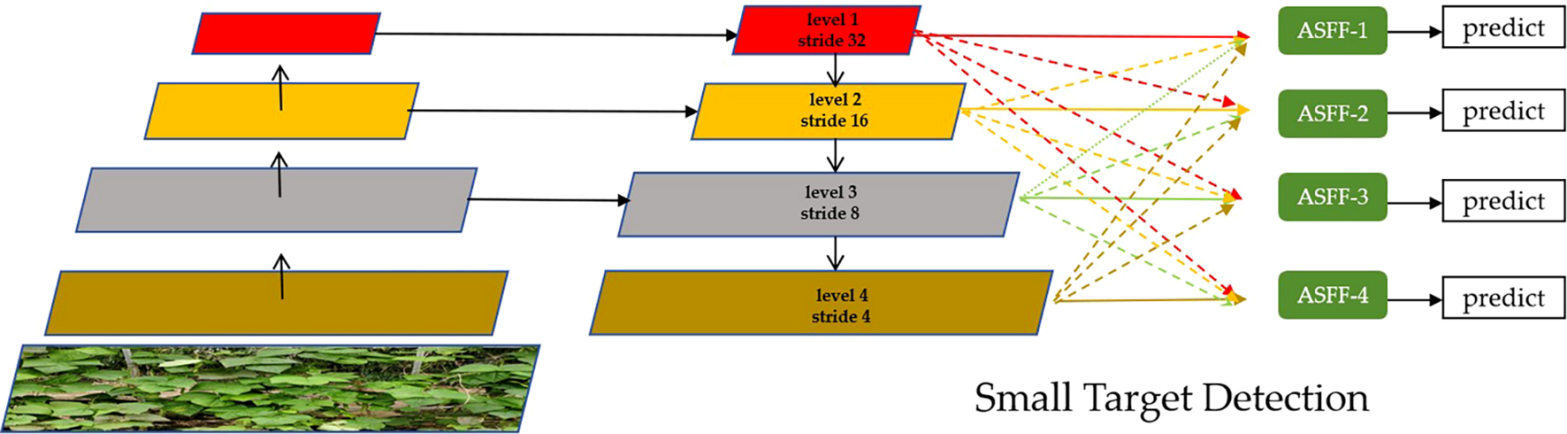
The improved SAFF fusion detection head structure principle.

Feature pyramid network (FPN) methods are commonly used to address scale variations in object detection tasks (Ghiasi et al., 2019). However, in agricultural scenes—particularly in open-field trellis cultivation environments—a single image contains diverse features, which limits the functionality of single-scale detectors based on FPN due to restrictions involving feature information. This study replaces the original feature fusion approach based on FPN with an ASFF method, enhancing detection performance. ASFF introduces an adaptive spatial feature fusion mechanism that effectively filters out irrelevant information, thereby improving scale invariance. Additionally, an improved ASFF specifically tailored for kidney bean object detection was implemented. This incorporated a fourth detection head for small target detection, forming the ASFF detection head. ASFF extracted feature maps from different stages of the backbone, denoted as P3, P4, and P5, which were responsible for detecting small, medium, and large objects, respectively. By incorporating learnable weights, it enabled each detection head to adaptively and selectively integrate feature information from other scales while mitigating inconsistencies such as semantic gaps, scale mismatches, and spatial misalignments across scales, thereby enhancing the robustness of multi-scale object detection. On this basis, an earlier and higher-resolution feature map was extracted from the backbone to construct a new detection head, P2, specifically designed for detecting extremely small objects. This P2 head retained richer details and higher spatial resolution. For the P2 detection head, its input features were derived from the same-level P2, the upper-level P3, as well as higher-level P4 and P5 (Huang et al., 2025). The structure principle of the improved ASFF mechanism is illustrated in **Figure 5**.

#### 2.2.5 Loss function Focaler-SIoU

**Figure 6:**
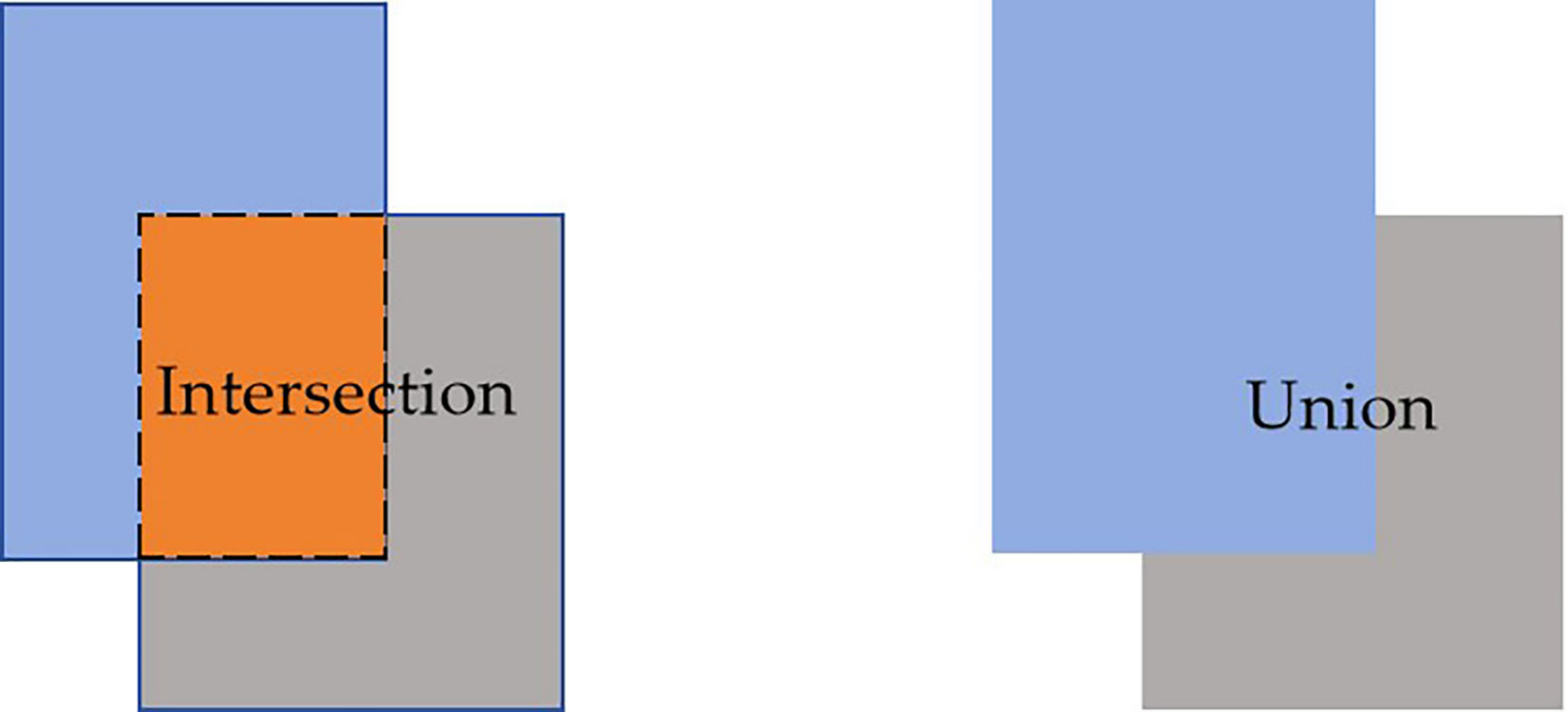
IoU principle.

In the natural environment, kidney bean fruit detection faces various challenges due to branch and fruit overlaps and different levels of occlusion. To address these challenges, we developed the Focaler-IoU algorithm and implemented a novel loss function module, Focaler-SIoU, to improve detection performance (Song et al., 2025). The Focaler-IoU algorithm focuses on different regression samples to enhance detector performance across various detection tasks. Through linear interval mapping, the Intersection over Union (IoU) loss is reconstructed to pay attention to different samples. This approach considers the distribution effects of difficult samples and simple samples on bounding box regression, a factor that is often overlooked in traditional IoU loss functions. The Focaler-IoU algorithm compensates for the limitations of existing bounding box regression methods through its unique approach, further improving detection performance across different tasks (Zhang and Zhang, 2024). Equation 2 defines Focaler-IoU, which adjusted the loss based on the value of the IoU, with the fundamental principle of IoU illustrated in **Figure 6**. When the IoU was less than a lower threshold d, the loss became 0; when the IoU exceeded an upper threshold u, the loss was 1; and when the IoU fell between d and u, the loss increased linearly as a function of the IoU value. This design allowed the loss function to remain sensitive to IoU values within a specific interval, thereby enabling a greater focus on samples where the predicted bounding box exhibited moderate overlap with the ground truth bounding box, i.e., samples that were neither excessively difficult nor overly easy. This facilitated improved learning of feature representations from these moderate samples rather than concentrating solely on the easiest or most challenging cases.

(2) $$\text{Io}{\text{U}}^{\text{focaler}}\{\begin{array}{c} 0,\;\;\;\;\;\;\;\;\;\;\;\;\;\;\;\;\;\;\;\;\;\;(\text{IoU}\;\;<\;\text{d})\;\\ \frac{\text{IoU}-\text{d}}{\text{u}-\text{d}},\;\;\;\;\;\;\;\;\;\;\;\;(\text{d}\ll \;\text{IoU}\;\ll \;\text{u})\\ 1,\;\;\;\;\;\;\;\;\;\;\;\;\;\;\;\;\;\;\;\;\;\;\;(\text{IoU}\;>\;\text{u}) \end{array}$$

The SIoU loss function was introduced by incorporating considerations of angles and scale sensitivity, thereby introducing a more complex boundary box regression method. This approach addressed the inadequacy of traditional loss functions in terms of adaptability in complex agricultural scene detection tasks, particularly in scenarios requiring multi-target small-object recognition. The SIoU loss function includes four components: angular loss, distance loss, shape loss, and an unspecified fourth component (Gevorgyan, 2022). By integrating these aspects, the method achieved improved training efficiency and prediction accuracy. The SIoU loss function is suitable for scenarios requiring precise boundary box alignment and is particularly applicable to the detection of kidney beans, which are relatively elongated objects. As Equation 3 demonstrates, it presents the detection principle based on SIoU.

(3) $$\text{SIoU}=\text{IoU}-{(0.5\times \text{(Shape cost}+\text{Distance cost)}+\text{eps)}}^{\alpha }$$

The Focaler-SIoU loss function was developed by balancing attention on diverse sample features and is particularly suitable for video screenshots. The loss function effectively addresses target detection for kidney beans—relatively thin objects—and the resolution variations inherent in screenshots.

### 2.3 Test platform setup

During the experimental process, we used servers for model training and optimization. We used Ubuntu 22.04 as the operating system, RTX4090D (24GB) GPU, 16 vCPU Intel(R) Xeon(R) Platinum 8481C CPU, and 80 GB of system memory. Python 3.12 was used as the programming language and PyTorch 2.3.0 as the deep learning framework.

### 2.4 Evaluation index

We adopted the following evaluation metrics: Precision (P), Recall (R), F1 score (F1), and average precision (AP). The AP value was calculated based on the IoU threshold of 0.5. The calculation methods for these metrics are detailed in Equations 4–7.

(4) $$\text{P}=\frac{{\text{T}}_{\text{p}}}{{\text{T}}_{\text{p}}+{\text{F}}_{\text{p}}}$$

(5) $$\text{R}=\frac{{\text{T}}_{\text{p}}}{{\text{T}}_{\text{p}}+{\text{F}}_{\text{N}}}$$

(6) $$\text{F}1=\frac{2\text{PR}}{\text{P}+\text{R}}$$

(7) $$\text{AP}=\int_{0}^{1}\text{P}(\text{R})\text{d}(\text{R})$$

where P represents the percentage of correctly identified kidney beans out of the total identified beans; R reflects the model’s detection ability for positive samples, representing the percentage of correctly identified kidney beans out of all kidney beans in the dataset; the F1 score reflects the balance between precision and recall, as well as the model’s ability to accurately identify most true samples while maintaining a low false positive rate; and AP represents the average precision across all detection results.

### 2.5 Experimental methods

To ensure sufficient training for the model, the experimental setup strictly followed a 7:2:1 ratio to partition the kidney bean dataset into training (2,818 images), validation (804 images), and test sets (404 images). This ensures the model can efficiently learn complex agricultural scenarios, particularly those involving open-field trellis cultivation environments, to capture the core features of kidney beans effectively.

The YOLO series includes different models developed for various application scenarios each tailored to different aspects such as lightness, accuracy, and robustness. These models have demonstrated strengths in various areas, including being lightweight, achieving high detection accuracy, and exhibiting robustness, while also showing limitations in terms of lightweight efficiency, detection precision, and robustness performance. In the context of agricultural applications, there is a growing emphasis on the deployment of lightweight detection models to achieve real-time processing while maintaining high detection accuracy. Of the common lightweight models in the YOLO series, such as YOLOv5n, YOLOv6n, YOLOv8n, and YOLOv10n, each exhibits distinct advantages in terms of resource consumption and computational efficiency. These models not only demonstrate low resource consumption and high computational efficiency but also exhibit outstanding performance in terms of accuracy and practicality in multi-target detection tasks, albeit with certain limitations in lightweight efficiency and real-world performance capabilities (Terven et al., 2023). Therefore, the study conducted experiments using YOLOv5n, YOLOv5n-p2, YOLOv5s, YOLOv6n, YOLOv10n, YOLOv10n-p2, and YOLOv9t on a self-built kidney bean dataset, comparing their performances with that of KidneyB-YOLO in two stages to confirm and validate the superior performance of KidneyB-YOLO in object detection tasks in agricultural environments.

To validate the performance differences of KidneyB-YOLO in actual scenarios, this study conducted experiments in which all models were trained from their initial states without loading pretrained weights from official releases. Though requiring more training time and computational resources, this approach was beneficial for ensuring the fairness, accuracy, and reliability of the experimental results. Realistically and objectively, the training process reflected how different models performed in kidney bean detection under open-field trellis cultivation scenarios. During the training process, input image sizes were uniformly scaled to 640×640 pixels, the batch size was set to 20, and the training epoch was set to 300 to ensure adequate model convergence. The final evaluation of model performance primarily utilized the AP metric with an IoU threshold of 0.50. ensuring thorough learning. The experimental results obtained were the basis for evaluating the performance of each model in terms of kidney bean detection in open-field trellis cultivation scenarios.

## 3 Results and discussion

### 3.1 Experiments with different detection methods

**Figure 7:**
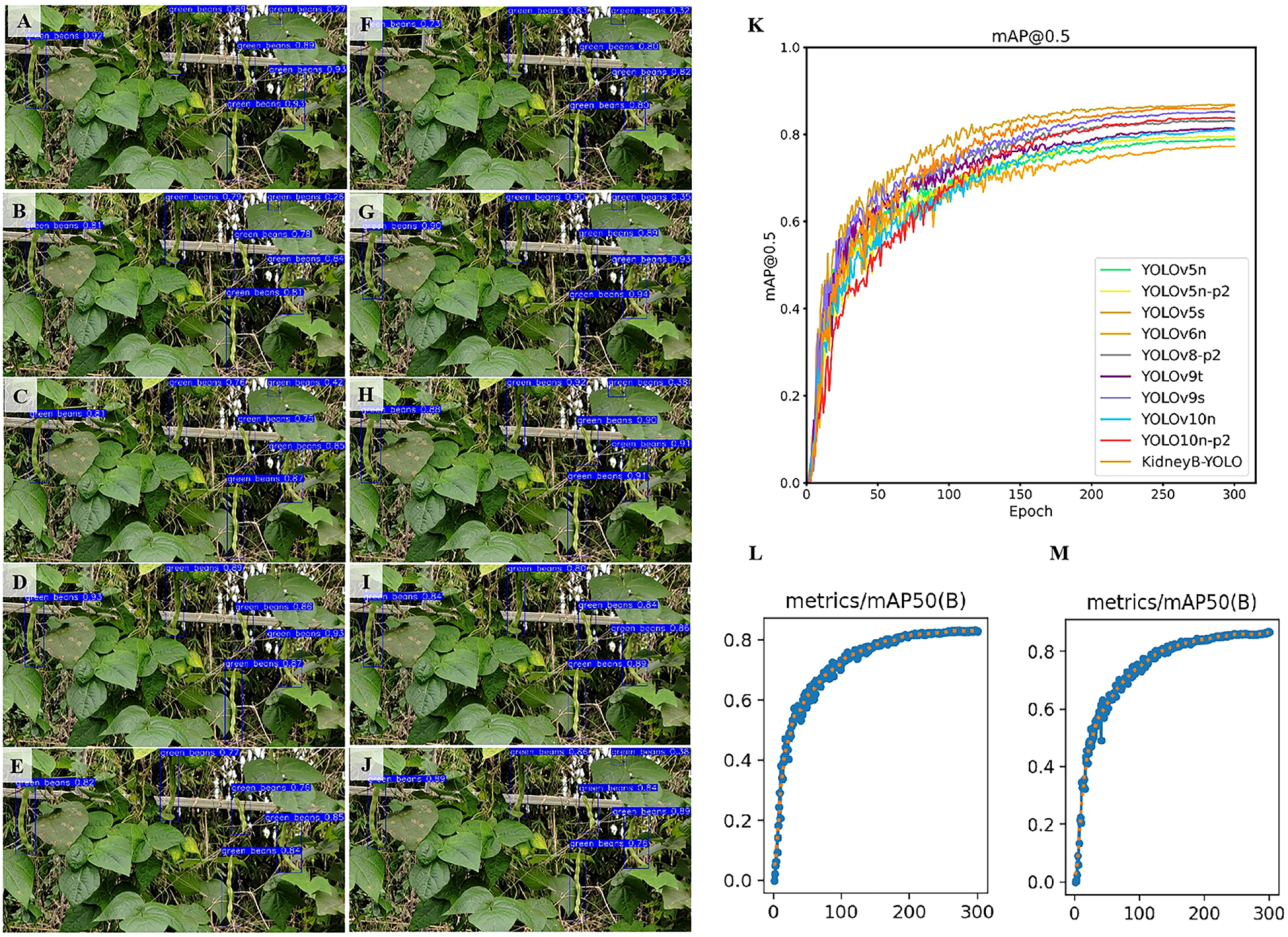
Performance comparison of different detection models on the kidney bean test set (Evaluation metric: mAP@0.5, Input size: 640×640) **(A)** KidneyB-YOLO, **(B)** YOLOv5n, **(C)** YOLOv5n-p2, **(D)** YOLOv5s, **(E)** YOLOv6n, **(F)** YOLOv9t, **(G)** YOLOv9s, **(H)** YOLOv10n, **(I)** YOLOv10n-p2, **(J)** YOLOv8n-p2, **(K)** Comparison of different models, **(L)** Original model, **(M)** Improved model.

To evaluate the performance of the KidneyB-YOLO model in detecting kidney beans, experiments were conducted using a variety of YOLO models, including YOLOv5n, YOLOv5n-p2, YOLOv5s, YOLOv6n, YOLOv8n-p2, YOLOv9s, YOLOv9t, YOLOv10n and YOLOv10n-p2, as demonstrated in **Figure 7**. Under scenarios of direct sunlight, inverse sunlight, and Gaussian blur, the KidneyB-YOLO model demonstrated high detection confidence without false negatives or false positives, thereby validating the improved model’s reliability and practicality in real-world scenarios.

The KidneyB-YOLO detection model possesses a file size of 12 MB and demonstrates superior AP metrics compared to the other models. **Table 1** demonstrates that, in terms of average precision, KidneyB-YYOLO outperforms the YOLOv5n, YOLOv5n-p2, YOLOv6n, YOLOv9t, YOLOv10n and YOLOv10n-p2 detection models by approximately 6.30%, 6.80%, 7.80%, 4.40%, and 2.70%, respectively. Specifically, in terms of detection accuracy for kidney bean fruits, we selected three models—YOLOv5t, YOLOv9s, and RT-DETR-X—as baseline models for comparison. The file sizes of these models were 17.70 MB, 14.50 MB, and 129.10 MB, respectively, all larger than that of the proposed KindyB-YOLO. The size of RT-DETR-X even exceeded that of KindyB-YOLO by more than tenfold. Among them, RT-DETR-X represented an end-to-end object detector based on the Transformer architecture.

**Table 1:** Performance comparison of different object detection models on the kidney bean dataset.

Model	Evaluation index						
	AP	P	R	Size	Parameters	GFLOPS	FPS
v5n	79.60%	79.70%	74.00%	5.0MB	2503139	7.1	58.3
v5n-p2	79.10%	78.60%	73.20%	5.0MB	2404676	10.8	28.8
v5s	85.80%	88.00%	80.20%	17.7MB	9111923	8.2	25.3
v6n	78.10%	80.40%	71.00%	8.3MB	4233843	11.8	64.1
v9t	81.50%	80.90%	76.20%	4.4MB	1970979	7.6	35.1
V9s	85.20%	83.10%	79.71%	14.5MB	7167475	26.7	23.1
v10n	80.40%	81.10%	73.80%	5.5MB	2694806	8.2	34.2
v10n-p2	83.20%	81.70%	77.50%	8.0MB	3894456	18.0	15.7
v8n-p2	82.50%	84.90%	77.20%	5.9MB	2921172	12.2	34.6
RT-DETR-X	87.30%	87.20%	81.20%	129.1MB	65469491	222.5	4.1
KidneyB-YOLO	85.90%	85.20%	79.90%	12.0MB	6058140	12.4	32.5

Although the YOLOv5s model achieved a notable AP of 85.80%, its parameter count increased by 211.93% compared to the baseline model YOLOv8n-P2, and its model size expanded by 316.07%. This indicates that YOLOv5s sacrifices lightweight structural efficiency for enhanced detection accuracy, yet its AP remains lower than that of KidneyB-YOLO. Similarly, the YOLOv9s model achieved an AP of 85.20%, with a size increase of 2.50 MB and a parameter count that expanded to 118.31% of the original, while its AP decreased by 0.70% compared to KindyB-YOLO. The RT-DETR-X model attained an mAP of 87.3%, yet the tenfold increase in model size resulted in only a 1.4% improvement in accuracy, which was deemed unacceptable. As the data indicated, the RT-DETR-X model achieved the highest detection accuracy 87.3% AP, exceeding our model by approximately 1.4%. However, this came at the cost of substantial computational overhead: its parameter count was nearly 10 times that of our model, its computational load GFLOPs was approximately 20 times greater, and its inference speed FPS was only 12.5% of ours.

Compared to the similarly P2-layer-enhanced YOLOv10n-P2 model, our model achieved a 2.7% higher mAP while reducing computational load by 31% and more than doubling the frames per second (FPS). This validated the effectiveness and efficiency of our improvement strategy.

Our model required 12.4 GFLOPs, which was higher than the most basic YOLOv5n (7.1 GFLOPs) but substantially lower than the performance-comparable YOLOv5s (23.8 GFLOPs). This indicated that the accuracy improvement achieved through introducing modules such as dynamic convolution and DAT came at a well-controlled and efficient computational cost.

The inference speed of our model reached 32.5 FPS, fully meeting real-time processing requirements (typically considered achievable above 30 FPS). Although this speed was lower than certain extremely lightweight models like YOLOv5n (58.3 FPS), the 6.3% improvement in accuracy meant this trade-off was fully justified. With a compact parameter size of only 12.0 MB, the model was particularly suitable for deployment on embedded devices and mobile robotic platforms with constrained computational and storage resources.

While the proposed model exhibits a slight increase in file size and parameter count relative to YOLOv8n-p2, the 12 MB size and approximately 6 million parameters fall within acceptable limits. By slightly increasing the model size and use of computational resources, KidneyB-YOLO achieves superior detection precision, making it a viable and practical choice for agricultural applications. For application scenarios such as agricultural harvesting robots that require real-time operation on embedded platforms, a model with 12 MB in size and 32.5 FPS demonstrated far more practical value than computationally intensive and slow models. This trade off underscores the effectiveness of the proposed model in balancing lightweight design with improved performance compared to conventional models.

### 3.2 The C2f_DynamicConv module’s effect

To validate the impact of the C2f_DynamicConv module on the performance of the YOLOv8n-p2 model, the DynamicConv module was integrated into the main backbone and neck modules of the YOLOv8n-p2 model by replacing the corresponding C2f modules. Three substitution patterns were implemented: (1) directly modifying the convolution blocks with the DynamicConv module, (2) integrating the DynamicConv module with the C2f module to form the C2f_DynamicConv module and replacing the main backbone with this module, and (3) replacing all the C2f modules in both the main backbone and neck with the C2f_DynamicConv module. The models were designated as YOLOv8n-p2-d1, YOLOv8n-p2-d2, and YOLOv8n-p2-d3, representing the first, second, and third substitution approaches, respectively. The detection performance of these models (which have the C2f_DynamicConv module incorporated at various positions) is illustrated in **Table 2**.

**Table 2:** Performance impact of integrating the C2f_DynamicConv module at different network locations.

Model	Evaluation index						
	P	R	F1	AP	Size	GFLOPS	FPS
YOLOv8n-p2	84.90%	77.20%	80.87%	82.50%	5.9 MB	12.2	34.6
YOLOv8n-p2-d_1_	83.30%	80.80%	82.03%	84.70%	12.6 MB	14.2	32.5
YOLOv8n-p2-d_2_	85.20%	79.90%	82.46%	85.90%	12.0 MB	14.1	32.5
YOLOv8n-p2-d_3_	85.60%	80.10%	82.76%	85.20%	10.6 MB	14.8	32.4

For the YOLOv8n-p2-d3 model, the integration resulted in a 1.8% improvement in F1 score and a 2.7% improvement in average precision compared to the original YOLOv8n-p2 model. Additionally, the model was less than double the size of the original model, maintaining a size of not over 12MB. These findings indicate that the C2f_DynamicConv module effectively enhances feature extraction, thereby improving detection accuracy and efficiency. However, the YOLOv8n-p2-d2 model, which achieved an average precision of 85.20% with a recall rate of 79.90%, showed limited improvement over the original model, with only a 2.60% enhancement in average precision and a 30.00% decline in F1 score compared to YOLOv8n-p2-d3. This suggests that replacing both the main backbone and neck networks with the C2f_DynamicConv module did not yield optimal results in terms of feature integration and detail extraction. On the other hand, the optimized YOLOv8n-p2-d1 model, which incorporated the C2f module with a focus on minimizing modifications, demonstrated enhanced performance with minimal changes to the model. Although this dynamic convolution model improved detection speed, it resulted in a significant increase in model size without a substantial improvement in aver-age precision. The performance differences between various models are illustrated in **Figure 7**. **Figure 7** provides a comparative analysis of the AP values across different models (K), with particular emphasis on the performance gap between the KidneyB-YOLO model (M) and the YOLOv8n-p2 model (L). Considering the balance between precision and compactness, the integration of the C2f_DynamicConv module into the main backbone of the YOLOv8n-p2 model not only enhances accuracy but also improves inference speed and efficiency.

### 3.3 Ablation experiment

Ablation experiments focus on the evaluation of different strategies to enhance target detection accuracy and speed, with the goal of optimizing the model and improving its performance in target detection (Karim et al., 2024). In this study, beginning with the YOLOv8n-p2 framework, the DynamicConv module was introduced to construct the C2f_DynamicConv module, replacing the C2f module in the main backbone. Additionally, an attention mechanism, referred to as DAT module, was incorporated, and modifications were made in the neck network. Simultaneously, the loss function was enhanced by replacing CIoU with Focaler-SIoU to validate the effectiveness of the proposed modifications. An improved feature fusion detection head, ASFF, was employed to address complex agricultural background scenarios for small target detection. To ensure the statistical reliability of the experimental results, each model configuration underwent three independent repeated training runs with different random seeds while maintaining identical training-validation dataset splits. The final performance metrics were calculated as the arithmetic mean of the three experimental results, supplemented by standard deviation calculations to evaluate model stability. To further quantify the statistical significance of performance differences, we performed a paired t-test comparing the final KidneyB-YOLO model with the baseline model (YOLOv8n-p2). The results demonstrated that the performance improvement was highly statistically significant (p-value< 0.001), confirming that the observed enhancements were not caused by random fluctuations. Through this series of systematic ablation studies, we conducted an in-depth analysis of the performance contributions from each improvement module and performed comprehensive comparisons with the baseline network. **Table 3** demonstrates that all key performance indicators exhibited standard deviations within 0.15%.The results demonstrated the effectiveness of the improved modules and their functional contributions. A comprehensive analysis of the ablation results is presented in **Table 3**.

**Table 3:** Ablation study results of KidneyB-YOLO: contribution evaluation of each improved module.

Model	Evaluation Index										
	DynamicConv	DAT	ASFFHead	FocalerSIoU	AP	P	R	F1	Size	GFLOPS	FPS
YOLOv8n-p2					82.50%	84.90%	77.20%	80.87%	5.9 MB	12.2	34.6
YOLOv8n-p2-C	✓				83.40%	84.80%	76.90%	80.66%	8.7 MB	10.8	32.9
YOLOv8n-p2-d		✓			82.60%	83.20%	77.10%	80.03%	6.5 MB	12.4	32.7
YOLOv8n-p2-H			✓		84.50%	84.90%	79.30%	82.00%	8.7 MB	12.3	32.6
YOLOv8n-p2-S				✓	82.70%	83.00%	75.70%	79.18%	5.9 MB	12.2	34.7
YOLOv8n-p2-dC	✓	✓			84.50%	84.10%	78.80%	81.36%	9.3 MB	11.1	33
YOLOv8n-p2-dCH	✓	✓	✓		85.60%	86.20%	79.20%	82.55%	12.0 MB	12.4	32.5
KidneyB-YOLO	✓	✓	✓	✓	85.90%	85.20%	79.90%	82.46%	12.0 MB	12.4	32.5

√ indicates that the model has added this module.

In the context of improving target detection accuracy in kidney bean detection, this research implements multiple improvement strategies aimed at enhancing the precision of small target detection for kidney beans. By employing the DAT attention mechanism, the model focuses on small regions of interest containing pertinent information while ignoring complex background interference. With a memory increase of 0.6MB, the AP improves by 0.1%. Although there is a slight decline in P, R, and F1, the overall performance shows improvement when combined with other modules. The incorporation of the DAT module resulted in an expansion of the model size, with only marginal improvements in accuracy. However, the integration of the DAT module enhanced the model’s autonomous ability to prioritize the most critical information, particularly in the context of kidney bean target recognition. This approach effectively addressed challenges such as significant scale variations between extremely large and small targets within the same image, clusters of multiple fruits, complex backgrounds, and the lack of prominent distinguishing features in kidney bean fruits. The introduced DAT module implemented a feature filtering mechanism within our model, which acquired a broader effective receptive field by predicting offset points. Replacing the C2f module in the main backbone with the C2f_DynamicConv module resulted in a 0.9% increase in AP, but with slight decreases in P, R, and F1. This study further validates the combined effect of the C2f_DynamicConv module and the DAT attention mechanism, demonstrating a significant enhancement in AP, R, and F, which increase by 2.0, 1.6, and 0.31 percentage points, respectively. Additionally, the incorporation of the improved ASFF head for feature fusion significantly improves detection precision, with the AP and R increasing by over 2% each. Finally, our research integrates the Focaler-SIoU optimization algorithm with the SIoU loss function, resulting in a slight improvement in AP without increasing model size. We also verified the performance of the KidneyB-YOLO model when the Focaler-SIoU loss function was removed and replaced with the original loss function, which demonstrated a notable improvement over the original model. The final performance of the KidneyB-YOLO model was an AP of 85.90%, a P of 85.20%, an R of 79.90%, and an F1 of 82.46%.

### 3.4 Validation of detection performance in complex agricultural backgrounds

**Figure 8:**
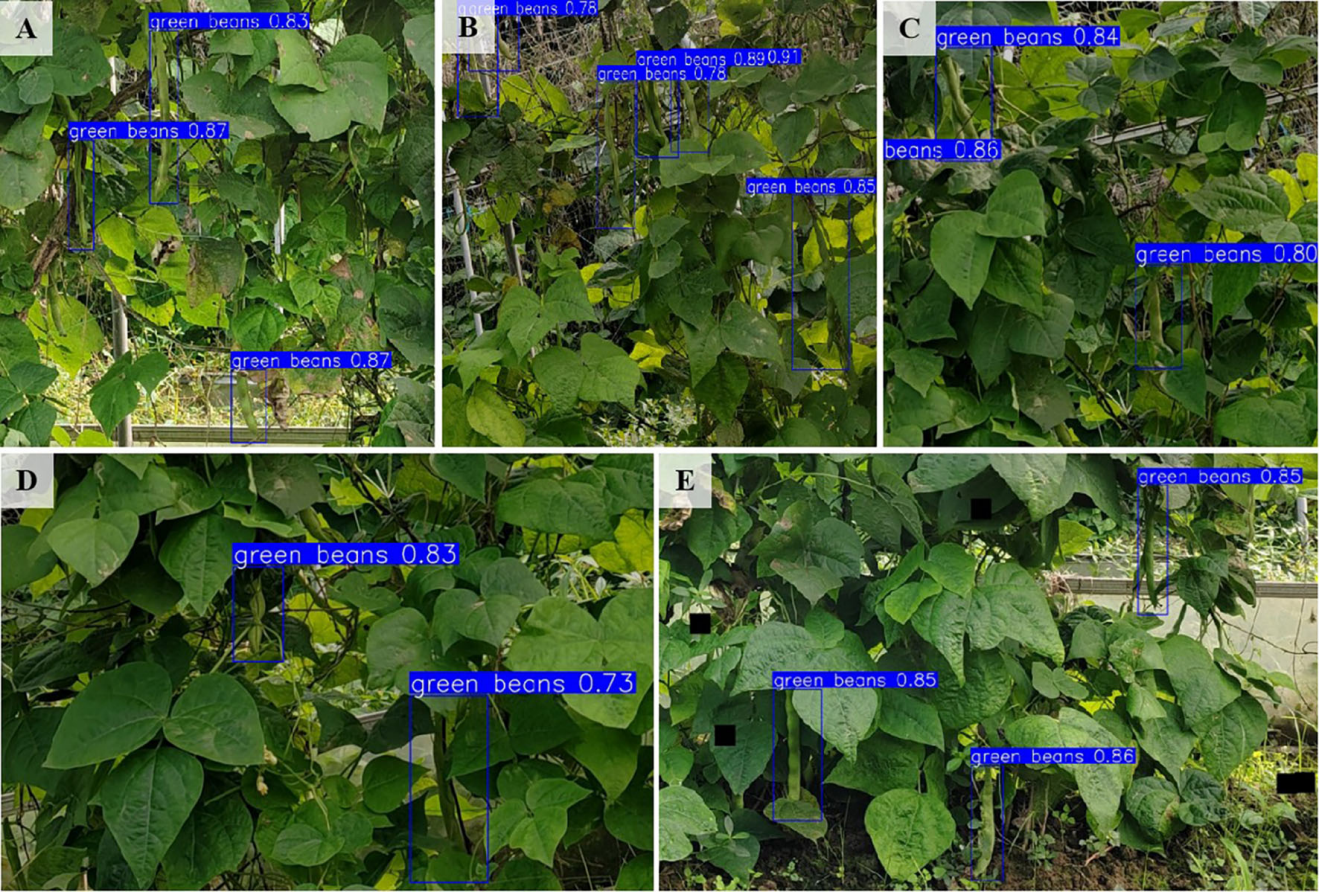
Detection results for the KidneyB-YOLO model in complex agricultural scenarios. **(A)** Multiple occlusions, **(B)** Fruit overlop, **(C-E)** Complex background.

To comprehensively evaluate the adaptability and generalization capabilities of the KidneyB-YOLO model in complex agricultural environments, this study captured images of kidney beans under various challenging conditions, including variations in light, background interference, leaf occlusion, individual occlusion of kidney beans, and size differences among fruits. These images were used for model detection comparisons. The results indicate that the improved model exhibits better performance when occlusion is severe; the other models display poor performance in this scenario. When multiple target detection is conducted, the improved model can detect small targets that the other models fail to identify; additionally, the improved model achieves high confidence in terms of average precision. **Figure 8** demonstrates that under conditions such as fruit occlusion (A), multiple fruit overlaps (B), and various complex background interferences (C) – (E), our model maintains a high average precision, with the detection accuracy reaching 91%. This demonstrates the detection improvement. The KidneyB-YOLO model exhibits strong robustness and generalization capabilities.

## 4 Discussion

This study developed the KidneyB-YOLO model, which could make a significant contribution to the identification and quantification of kidney beans. The model employs improvement approaches that can adaptively handle images with varying resolutions, complex backgrounds, and different formats, including the DynamicConv module, a DAT, and the Focaler-SIoU loss function, which is tailored for different sample features. Although these modifications slightly increase the model’s parameters and dimensions, they significantly improve the precision of kidney bean detection in open-field trellis cultivation scenarios. Kidney beans cultivated under open-air trellises exhibited substantial variations in size, orientation, and occlusion levels. Convolutional kernels with fixed parameters demonstrated limited generalization capability. DynamicConv module enabled the model to adaptively adjust its parameters based on input images, allowing it to generate more discriminative features for diverse kidney bean targets. This approach enhanced the model’s robustness to scene variations. To address the challenges posed by significant scale and pose variations of targets as well as complex backgrounds, dynamic convolution was introduced. Under open-air cultivation conditions, kidney beans and leaves exhibited similar colors, and the beans themselves had slender, elongated shapes. Traditional attention mechanisms were often distracted by large areas of homogeneous background. The deformable attention mechanism in DAT adaptively concentrated computational resources on key regions in the image. For slender bean pods, it learned to “project” attention onto the target contours, effectively extracting features from complex backgrounds and reducing missed detections. We introduced the DAT mechanism to address the issues of color similarity between targets and background as well as the elongated structure of the targets. Kidney beans represent typical small objects, whose characteristic features tend to be lost in deeper network layers. Direct fusion of multi-level features often resulted in semantic conflicts. We adopted ASFF to adaptively filter out conflicting information within the feature pyramid and incorporated an additional detection layer specifically designed for small targets. This design ensured that fine-grained features of kidney beans extracted from feature maps could be harmoniously integrated and utilized for final prediction, directly addressing the morphological characteristics of kidney beans. An improved ASFF module along with a dedicated detection layer was designed to tackle the challenges of small object detection and feature conflict. The dataset contained a substantial number of samples with varying degrees of occlusion and scale differences caused by varying shooting distances, which accurately reflected real-world acquisition conditions. Focaler-SIoU integrated the direction-aware property of SIoU with the hard sample focusing mechanism of Focaler-IoU, guiding the model to prioritize the optimization of hard-to-recognize kidney bean samples during training, thereby improving overall generalization performance. The Focaler-SIoU loss function was introduced to address the issues of non-uniform sample resolutions and the imbalanced distribution of easy and hard samples. Our objective was to elucidate the synergistic mechanisms of these modules in addressing specific agricultural vision tasks. Dynamic convolution provided a rich set of adaptive features. DAT subsequently performed a “refinement” process on these features, accurately identifying and selecting the key characteristics of kidney beans. ASFF, combined with the dedicated detection layer, ensured that these refined features were fused without degradation and utilized for small target prediction. Focaler-SIoU guided the entire system to focus on hard examples. We adopted a method aiming to achieve a significant improvement in precision without substantially increasing computational load, a method differing from those in the existing literature. This method, while diverging from traditional approaches, demonstrates enhanced precision, highlighting the model’s robustness and adaptability (Shafiee et al., 2017; Shao et al., 2022; Zhai et al., 2025; Zhao et al., 2025). Studies emphasize the importance of a lightweight design in model structures, but there may be limited improvement in detection accuracy while reducing computational complexity and parameter count. The integration of the DAT mechanism markedly enhanced the model’s ability to suppress complex background interference and improved the discriminative power of feature extraction. It is worth emphasizing that adding the DAT module alone provided limited direct gains in accuracy and introduced a certain increase in parameters, underscoring that the core of deep learning model architecture lies in the synergy among modules and systemic balance.

The essential function of the DAT module is that of a feature filter. When the raw features extracted by the backbone network were of low quality, the DAT module was unable to accurately identify salient image information, potentially misallocating attention to background noise or less relevant regions. However, the role of the incorporated DAT module extended far beyond the current detection task: it significantly strengthened the model’s capacity for multi-scale feature and spatial contextual modeling. This enhancement was intended to facilitate the future evolution of the model from bounding-box-level to pixel-level prediction, thereby improving contextual understanding and multi-scale feature integration capabilities to capture richer spatial information.

Acting as a smooth transitional bridge, the DAT module adaptively focused on detail-rich key points and integrated global contextual information to refine local features. By dynamically generating sampling points with varying distributions and densities for objects of different scales, the module enabled the backbone network—already equipped with multi-scale perception—to seamlessly adapt to dense prediction tasks such as semantic segmentation without major structural modifications. This capability allowed the model to effectively handle object regions of vastly different sizes in segmentation tasks, thereby eliminating the need to redesign or retrain a dedicated multi-scale segmentation module. This design reflects the foresight of our approach in terms of structural scalability.

Our work did not aim to invent fundamentally new algorithms, but rather constituted a “problem-driven algorithmic adaptation and integration innovation.” In the applied research context, we addressed a highly complex and underexplored challenge—detecting kidney beans under open-air trellises—by intelligently selecting, refining, and synergistically integrating state-of-the-art components to construct an efficient and effective system that surpassed baseline models.

Regarding the use of Dynamic Convolution, unlike its conventional application, our ablation studies revealed that placing it in the neck network yielded the most significant accuracy improvement. This was because the neck handles multi-scale feature fusion, and introducing dynamic adaptability at this stage enabled the model to more effectively integrate kidney bean features across different scales—particularly small-scale characteristics—representing a design decision tailored to the multi-scale nature of kidney beans.

The DAT mechanism served as a precise feature extractor for elongated small targets. We innovatively leveraged its deformable attention to address the distinctive elongated morphology of kidney beans. By learning offset values, the attention windows could distribute along the pods’ orientation, providing a more targeted approach than conventional rectangular attention mechanisms.

In constructing the synergistic architecture of “ASFF add dedicated small-object detection layer,” we utilized ASFF to resolve feature conflicts while adding a specialized detection layer for small targets. This design directly addressed the small size of kidney beans, complementing ASFF to ensure the preservation and effective utilization of subtle features—an innovation oriented toward the specific problem.

We built a coordinated pipeline design encompassing “ Dynamic Conv, DAT, ASFF, and Focaler-SIoU.” This constituted the key distinction between our model and YOLO variants that merely combine multiple modules. We thoroughly validated that incorporating this series of adaptive modules proved effective for detecting crops with specific morphological traits like kidney beans. This work offers a valuable improvement paradigm and technical reference for other analogous agricultural object detection tasks—such as detecting cucumbers and peppers—that face challenges including severe occlusion, color similarity, small target size, and variable sample resolutions.

In this study, the developed KidneyB-YOLO model demonstrated significant performance in kidney bean recognition and quantification; however, like any technical solution, it possesses certain limitations. These limitations highlight valuable directions for future research. The current work primarily focused on bounding-box-level object detection. In complex agricultural environments, occlusion—both by leaves and between fruits—is prevalent. The model’s capability to detect severely occluded targets remains suboptimal, occasionally leading to missed detections or reduced localization accuracy for partially occluded objects. Future investigations could explore the following avenues to mitigate occlusion-related issues, introducing a multi-view image acquisition system. Multi-view fusion strategies could construct a more comprehensive feature representation of objects, compensating for information loss in two-dimensional imagery through spatial information integration. Further optimizing the attention mechanism building upon the existing DAT module, enabling it to more precisely focus on visible parts of targets and enhancing the model’s capacity for dynamic feature extraction of occluded objects. Although KidneyB-YOLO excelled in object detection tasks, the current model does not support instance segmentation. Despite attempts to construct an object recognition model with a DAT module, it did not achieve the requirement for providing pixel-level precise contour information necessary for instance segmentation. For future extension to instance segmentation tasks, we considered replacing the detection head with a lightweight segmentation head on the existing DAT-equipped backbone and introducing loss functions specifically optimized for segmentation tasks to form a new semantic segmentation model. Since the backbone has been well-trained on detection tasks and possesses the robust capabilities of the DAT module, the model is expected to converge rapidly and start with a high performance baseline.

In summary, based on the YOLOv8-P2 framework, the KidneyB-YOLO model demonstrates outstanding performance in identifying kidney bean fruit targets. Our experimental results validated the model’s precision, providing reliable solutions to enhance the accuracy and efficiency of agricultural operations. The findings of this study not only validate the model’s applicability to real-world applications but also provide a foundation for the development of similar models in the future. As the demand for high-performance intelligent agricultural equipment grows increasingly urgent (Hu et al., 2020; Liu et al., 2021), the need for innovative solutions like the KidneyB-YOLO will increase, pushing fruit target recognition toward more efficient and intelligent approaches.

## 5 Conclusions

This study successfully constructed a kidney bean object detection model named KidneyB-YOLO for complex open-air trellis environments. By incorporating the C2f_DynamicConv module, DAT attention mechanism, an improved ASFF method, and the Focaler-SIoU loss function based on the YOLOv8n-p2 baseline, the model significantly increased the AP by 3.40 percentage points to 85.90%, while maintaining a lightweight size of 12 MB and real-time performance of 32.5 FPS. The improved model, referred to as KidneyB-YOLO, achieved an AP of 85.90%, which is 3.40% higher than the baseline YOLOv8-P2. The final model, KidneyB-YOLO, demonstrated robust performance in complex agricultural environments, achieving efficient and accurate detection of kidney bean crops and demonstrating good adaptability to unknown scenarios. Ablation studies revealed a key finding: individual improvement modules (such as DynamicConv or DAT) provided limited performance gains or even caused fluctuations in other metrics when introduced separately. However, when they worked collaboratively, a substantial performance leap was achieved. The underlying mechanism for this phenomenon lies in the fact that the modules formed a problem-oriented, complementary enhancement loop. Specifically, DynamicConv enhanced the model’s ability to extract basic features from multi-scale and multi-form kidney beans through its parameter adaptation capability, thereby providing richer feature maps for subsequent processing. Building upon this, the DAT attention mechanism utilized its deformable attention field to precisely focus on key feature regions of slender kidney beans, effectively suppressing interference from complex backgrounds. The improved ASFF structure and dedicated detection layer optimized the feature flow by resolving conflicts among multi-scale features through an adaptive fusion strategy and ensuring the full utilization of small object features, thereby systematically consolidating the gains from the aforementioned modules. The Focaler-SIoU loss function performed global optimization by focusing on hard examples and refining bounding box regression direction, guiding the entire model to concentrate its learning capacity on addressing challenging cases such as occlusions and deformations. The performance improvement did not result from a simple additive effect, but stemmed from the synergistic interaction of these modules across the complete pipeline of “feature enhancement, feature selection, feature fusion, and loss optimization.” Together, they constructed a fully adaptive detection system, endowing it with strong robustness for complex agricultural scenarios.

Future research work will focus on instance segmentation tasks for kidney beans in complex agricultural environments. Furthermore, we will precisely analyze the individual growth status of kidney beans in agricultural scenarios, including determining whether they are infected with diseases or pests, the type of disease or pest, and whether fertilization meets needs of kidney beans at different growth stages, to enable precise adjustment of their growth environments. Additionally, future work will focus on predicting the growth trends of kidney beans, such as their shape and yield, to provide precise adjustments for their growth environments.

## Data Availability

The original contributions presented in the study are included in the article/supplementary material. Further inquiries can be directed to the corresponding author.
